# The Evolution of Functional Amyloids and Their Impact on Host–Microbe Interactions

**DOI:** 10.1002/advs.202510109

**Published:** 2025-09-03

**Authors:** Divya Kolli, Saroj K. Rout, Roland Riek, Matthew R. Chapman

**Affiliations:** ^1^ Department of Molecular, Cellular, and Developmental Biology University of Michigan Ann Arbor MI 48109 USA; ^2^ Institute of Molecular Physical Science Swiss Federal Institute of Technology ETH Hönggerberg Zurich 8093 Switzerland

**Keywords:** biofilm, curli fiber, functional amyloid, host‐microbiome, origin of life

## Abstract

Amyloids are highly ordered β‐sheet‐rich structures that are well conserved across the domains of life. Amyloids have a unique repetitive structure that enables autocatalytic self‐replication. This property is most well‐known in the context of neurodegeneration, in which proteins misfold into amyloid and begin an amyloid cascade resulting in the deposition of large amyloid aggregates characteristic of various diseases such as Alzheimer's disease and Parkinson's disease. The amyloid fold, however, can be pathological or functional. The repetitive nature of amyloids positions self‐replicating amyloids as a potential key player in the origin of life. This may explain why, despite the pathogenic potential of amyloids, the amyloid fold is readily found. Many amyloids are not pathogenic and instead they contribute positively to the overall fitness of the cell. Bacteria, for example, use functional amyloids to facilitate biofilm formation, dissemination, storage, adhesion to cells or surfaces, and virulence. Interestingly, the high conservation of the amyloid fold and its ability to self‐replicate enables bacterial functional amyloids to accelerate amyloid‐associated disease in a human host. Here, the structure, conservation, and biology of the bacterial functional amyloids, as well as their impact on human health, are discussed.

## Graphical abstract

This review describes functional amyloids and highlights the importance of structural repetition and molecular mimicry that distinguish the amyloid fold. The authors describe how amyloid can be the last universal common ancestor of protein folds. The authors also discuss how microbes utilize functional amyloids and how the structural characteristics of these proteins aid in bacterial infection.

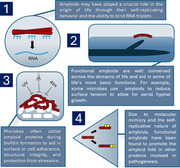

## 1 Introduction: The Amyloid Fold and Its Properties

Amyloids are composed of numerous copies of a peptide or protein, typically ranging from hundreds to thousands. While a single peptide or protein is usually involved, there are cases where pairs or triplets contribute to amyloid formation. These molecules are arranged in a highly ordered, repetitive, one‐dimensional manner, forming unbranched fibrils extending several micrometers in length.^[^ ^1^ ^]^ The fundamental repeating substructure, known as the cross‐β‐sheet motif, consists of two layers of intermolecular β‐sheets aligned along the fibril axis. The β‐strands run perpendicular to the fiber axis to form sheets that are often densely packed through the interdigitation of the side chains to form a motif which has been referred to as a steric zipper.^[^ ^2^, ^3^, ^4^, ^5^ ^]^ The interface of the sheets is analogous to the interior of a protein and forms the hydrophobic core, which is often devoid of water. The β‐sheets can adopt either a parallel or antiparallel arrangement, typically in register. In a parallel β‐sheet, each β‐strand lies directly above the one below it at a distance of 4.8 Å, whereas in an antiparallel sheet, each β‐strand aligns with the strand two rows beneath it at a distance of 9.6 Å. So far, antiparallel structures have only been observed in peptides. Amyloids formed by full‐length proteins exhibit greater structural complexity, often incorporating multiple cross‐β‐sheet motifs.^[^ ^6^, ^7^, ^8^, ^9^, ^10^ ^]^ The amyloid is a highly stable structure due to the contribution of the H‐bonds that point up and down the β‐sheets, the van der Waals forces between two interacting sheets, the salt bridges between sidechain amino acids, and the π–π interactions of the aromatic residues.^[^ ^11^, ^12^ ^]^

The amyloid structure is associated with unique properties,^[^ ^13^ ^]^ suggesting a wide array of functions. These functional amyloids can be categorized into three main types:^[^ ^14^ ^]^ (i) chemical storage, (ii) structural support, and (iii) information carrier. Amyloid formation occurs through a two‐state transition, shifting from a soluble (monomeric) state to a solid (amyloid) state, which involves a transformation from a flexible peptide or protein into a highly structured entity that is influenced by the buffer environment. The intermolecular arrangement characterized by the cross‐β‐sheet motif facilitates the concentration‐dependent folding.

The repetitive nature of amyloids provides a structural template for replication, forming the basis of the prion transmission mechanism through the dissemination of small fibril fragments.^[^ ^15^, ^16^, ^17^ ^]^ This repetitiveness can thereby also function as an oligomerization domain often key for biological signaling, as illustrated by the HET‐s/HET‐S prion system, where oligomerization triggers limited cell death, resulting in functional heterokaryon incompatibility.^[^ ^15^ ^]^ The repetitiveness occurs at the sub‐nanometer level, allowing for a repetitive arrangement of identical side chains that can confer novel binding specificities absent in the monomeric state. For instance, a single amphipathic β‐strand with positively charged side chains would display minimal affinity for negatively charged membranes or RNA, but an amyloid fibril composed of this peptide could bind tightly to both lipids and RNA molecules.^[^ ^18^ ^]^ Indeed, functional amyloids that play critical roles in surface interactions are well documented, including hydrophobins^[^ ^19^, ^20^, ^21^ ^]^ and bioemulsifiers.^[^ ^22^ ^]^

The amyloid surface exhibits cooperativity or avidity effects, characterized by the repetitive display of binding pockets. This feature enhances the overall apparent binding of a ligand, allowing it to diffuse from one binding site to another, leading to a very low overall dissociation rate. In fact, functional amyloids in bacterial biofilms have been proposed to act as sponges or reservoirs, effectively retaining small quorum‐sensing molecules within the biofilm by providing an extensive (albeit low‐specificity) binding surface.^[^ ^23^ ^]^ Furthermore, Pmel17 amyloid templates play a role in the synthesis of melanin within melanosomes involved in pigmentation.^[^ ^24^, ^25^ ^]^ In addition, amyloid segments may be used as oligomerization domains within a larger protein. The amyloid may thereby silence the protein/ peptide function^[^ ^26^ ^]^ or/and store the protein at high density inertly because of the high stability of the amyloid both under harsh chemical conditions as well as under biological proteolysis.^[^ ^27^, ^28^ ^]^ Since the amyloids often consist of ultra‐stable fibrils measuring several micrometers in length, which can integrate into mesoscopic and macroscopic structures, including hydrogels, their structure‐supporting role is evident. These include not only the biofilm formation described in detail below but also an extracellular amyloid matrix that surrounds, for example, an oocyte.^[^ ^29^, ^30^ ^]^

In this review, we focus on the evolutionary history and subsequent usage of the amyloid fold. We highlight how the unique properties and repetitive nature of the amyloid structure position amyloid sequences as potential drivers in the origin of life. In addition, we discuss how the high conservation of these amyloidogenic sequences and their potential role in the origin of life may identify amyloids as the common ancestor of protein folds. Finally, we propose that the structural and evolutionary conservation of amyloids is imperative to understanding and studying the multifaceted role that functional amyloids play in microbial–host interactions.

### 1.1 Functional versus Disease‐Associated Amyloid Fibrils

Amyloids were first identified and characterized in the context of diseases. They are associated with a couple of dozen age‐related human conditions, including Parkinson's disease, Alzheimer's disease, and type II diabetes^[^ ^9^, ^13^, ^31^ ^]^ and may also play a role in cancer.^[^ ^32^ ^]^ In these diseases, insoluble amyloid aggregates accumulate extra‐ or intracellularly in tissues and organs. In all disease cases, aggregation is attributed to the misfolding of a specific protein. A key question is why some amyloids are associated with diseases, while others are functional, given that functionality must be regarded as context‐dependent. This question is challenging to answer, partly due to a limited understanding of the diverse toxic mechanisms related to the aggregation kinetics of disease‐associated proteins. There are open questions regarding whether oligomers or amyloids serve as toxic agents.^[^ ^33^, ^34^, ^35^, ^36^, ^37^ ^]^ Furthermore, it remains unclear whether the aggregation process itself is toxic or simply a structural form of the protein.^[^ ^38^, ^39^, ^40^ ^]^

The quest to understand toxicity in amyloids may stem from (i) the distinct structural features of the amyloids or (ii) the aggregation kinetics and cellular influence and control thereof. Evidence supporting the latter is based on the observation that functional amyloids can form significantly more quickly than those associated with disease. For instance, the fruit fly protein Orb2 forms functional amyloids within a few minutes, reducing the chance of accumulating potentially toxic intermediates.^[^ ^41^ ^]^ Moreover, it appears that the functional amyloids aggregate kinetically by primary nucleation (i.e., the critical step is the formation of a nucleus), while secondary nucleation on the fibril surface is key in pathological amyloids.^[^ ^42^, ^43^, ^44^, ^45^ ^]^ While the proteostasis machinery of the cell can likely control primary nucleation, secondary nucleation is regarded as challenging because of its autocatalytic nature.^[^ ^43^, ^44^, ^45^, ^46^ ^]^ Indeed, designated chaperones are known to inhibit the periplasmic aggregation of bacterial functional amyloid CsgA/FapC^[^ ^47^, ^48^ ^]^ and human functional amyloid Pmel17 aggregation involved in pigmentation is controlled by many levels, including passage through multivesicular, endosomal compartments and proteolytic processing by a proprotein convertase.^[^ ^49^, ^50^ ^]^ An alternative hypothesis states that oligomers of functional amyloids may be less toxic than oligomers from disease‐associated amyloids. While there is limited scientific support for this question, it can be stated that not all amyloid oligomers are toxic, including those from Abeta.^[^ ^51^ ^]^

**Figure 1:**
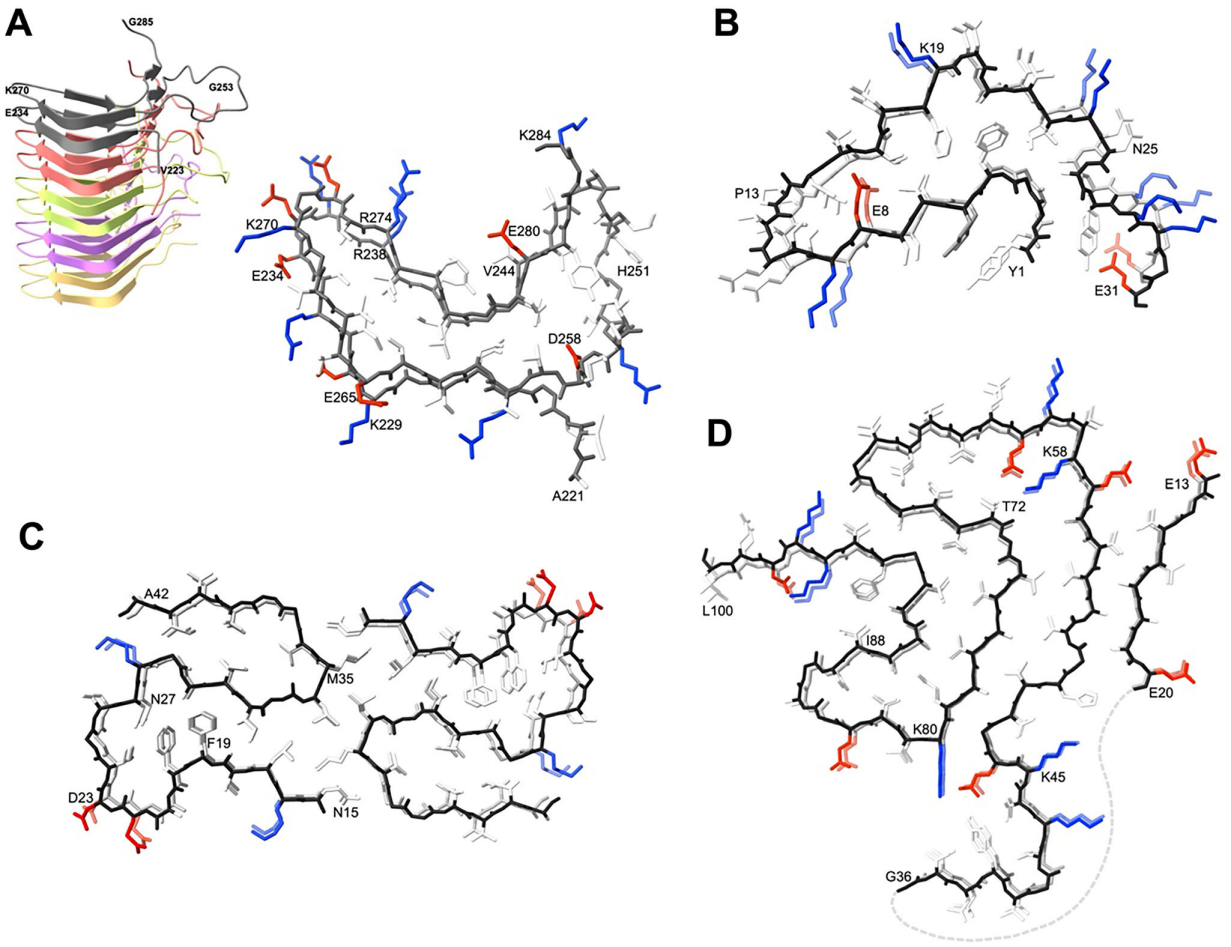
3D structures of A,B) functional and C,D) disease‐associated amyloids. The backbone of the top layer is shown in black. Side chains of K,R) positively charged residues are shown in blue, D,E) negatively charged residues in red, and all other residues in white. All structures are composed of a hydrophobic core and a hydrophilic surface, but there are structural differences between functional and disease‐associated fibrils as discussed in the text. A) Solid‐state NMR structure of the HET‐s(218–289) prion (PDB: 2RNM). Each HET‐s molecule forms two intramolecular windings within a β‐solenoid fold, which assemble into a repeating intermolecular structure. The left panel shows a side view of five stacked molecules in cartoon representation, colored distinctly. The top molecule (gray) is shown in detail (right) with side chains visible, viewed along the fibril axis. Adapted with permission.^[^ ^6^ ^]^ Copyright 2008, AAAS. B) Solid‐state NMR structure of β‐endorphin functional amyloid (PDB: 6TUB), viewed along the protofibril axis. Two molecules are shown. Glutamate 8 (E8) serves as a pH‐sensitive switch that enables reversible disassembly essential for biological function. Adapted with permission.^[^ ^53^ ^]^ Copyright 2020, The Authors. C) Solid‐state NMR structure of the Alzheimer's disease‐associated Aβ(1–42) fibril (PDB: 2NAO), viewed along the fibril axis. The amyloid core comprises residues 15–42 and displays C2 symmetry. Each protofilament in the steric zipper consists of two layers of intermolecular β‐sheets. Adapted with permission.^[^ ^8^ ^]^ Copyright 2016, The Authors. D) Cryo‐EM structure of the Type 1M polymorph of α‐synuclein fibrils (PDB: 8PK2), providing structural insights into juvenile‐onset synucleinopathy. A single protofilament is shown, highlighting two layers of intermolecular β‐sheets, viewed along the fibril axis. The dashed line marks the intervening residues. Adapted with permission.^[^ ^55^ ^]^ Copyright 2024, The Authors. Refer to Frey et al. (2024)^[^ ^55^ ^]^ for other reported polymorphs. Selected residues are labeled using the one‐letter amino acid code and residue number.

There are notable differences between disease‐associated and functional amyloids. Arguably, the biggest difference is the simplest—disease‐associated proteins did not evolve to form amyloid structures, whereas functional amyloids have. For instance, the HET‐s prion system is established through a gene duplication event that neutralizes surface charges, resulting in two layers per molecule along the fibril structure (**Figure** **1**A).^[^ ^6^, ^15^ ^]^ In addition, β‐endorphin fibrils contain a protonated Glu8 residue at their core, which acts as a pH trigger, facilitating the transition from the acidic phosphate‐containing environment of secretory granules (pH 5) to the neutral phosphate‐free environment of blood, thus allowing the fibril to dissolve (Figure 1B).^[^ ^52^, ^53^ ^]^ Thereby, the positively charged Lys residues are also regarded to play an important role in dissolution as the negatively charged phosphate buffering the Lysine repulsion in the secretory granules having a stabilizing effect that is absent in the blood. In contrast, disease‐associated fibrils, such as Aβ(1–42), tau, and α‐synuclein amyloid structures, contain frustrated structural segments. In protein folding frustration is a structural segment that is not energetically minimized.^[^ ^54^ ^]^ As the amino acid sequences did not evolve for the specific structural states they are thus compelled into energetically unfavorable conformations.^[^ ^54^ ^]^ For the Aβ(1–42) polymorph shown in Figure 1C, the backbone structure from F19 to D23 exhibits steric backbone frustration having F19 and F20 side chains facing the core and A21–D23 side chains facing the solvent (Figure 1C).^[^ ^8^ ^]^ The α‐synuclein fibril polymorph of Figure 1D features a hydrophilic core with a central void, a salt bridge in the core, and two spatially proximate Lys residues alongside hydrophobic surfaces.^[^ ^55^ ^]^ The tau fibrils exhibit numerous polar and charged interactions within their core structure, along with a relatively large surface‐to‐volume ratio.^[^ ^10^ ^]^ It is also noted that each polymorph may show distinct frustrations. It is hypothesized that frustrated regions and hydrophobic surface patches can be recognized by chaperones or other elements of the proteostasis machinery, such as nascent chain‐binding proteins. This interaction may lead amyloid structures to titrate out the available proteostasis proteins in unproductive binding complexes, with predictably detrimental effects on cellular proteostasis.^[^ ^56^ ^]^ Alternatively, hydrophobic patches or fuzzy tails that are composed of intrinsically disordered segments of the protein decorating the surface of the fibril core might play a crucial role in the secondary nucleation mechanism indicated earlier.^[^ ^42^ ^]^

Another level of structural complexity is associated with disease‐related amyloids known as fibrillary polymorphism,^[^ ^57^ ^]^ the existence of multiple structurally distinct amyloid states for both Aβ^[^ ^58^ ^]^ and α‐synuclein.^[^ ^55^ ^]^ This variability is in stark contrast to functional amyloids, which generally exhibit a single monomorphic structure at least under physiological conditions and occasionally even under more extreme conditions.^[^ ^53^, ^59^ ^]^ This trend is to be expected under the assumption of sequence optimization. The polymorphic nature of the disease‐associated fibrils is particularly fascinating. For instance, a slight change in pH can alter the polymorphisms, as demonstrated for α‐synuclein,^[^ ^55^ ^]^ or a change in salt composition can yield a different tau amyloid polymorph.^[^ ^10^ ^]^ This chameleon‐like property suggests many distinct structural polymorphs with similar energies, indicating a broad range of low‐energy minima. This behavior is unexpected for functional amyloids unless it serves a particular function or the amyloid function is polymorph unspecific. Moreover, each disease phenotype exhibits distinct structural states, as shown in tauopathies and synucleinopathies.^[^ ^60^, ^61^, ^62^ ^]^ In addition, reproducing these disease‐associated polymorphs in vitro appears to be quite challenging. Currently, only a few disease‐relevant polymorphs have been successfully reconstituted in vitro: the tau Alzheimer polymorph,^[^ ^10^ ^]^ Aβ(1–42) with a reported RMSD of ≈3 Å to the in vivo derived polymorph (Figure 1C)^[^ ^8^ ^]^ and the juvenile‐onset synucleinopathy polymorph (albeit missing one adjacent strand) (Figure 1D).^[^ ^55^ ^]^ While this suggests that the presence or absence of polymorphisms may help distinguish functional amyloids from disease‐associated amyloids, comprehensive in vivo structural data on functional amyloids is necessary to draw a definitive conclusion.

### 1.2 On the Potential Role of Functional Peptide Amyloids in the Origin of Life

Life is a complex phenomenon resulting from dynamic and multifaceted interactions among various molecules, primarily nucleic acids, proteins, and lipids. To understand the origin of life, it is essential to explore synthetic pathways that produce these molecules and to examine how their functional interplay resists thermodynamic equilibrium. Ultimately, these fundamental building blocks integrate into a unified system, forming a mutualistic relationship that harnesses an energy gradient for sustenance. One of the most widely accepted and prominent frameworks for the origin of life is the RNA world hypothesis, which is inspired by RNA's unique ability to function both as a catalyst and a genetic carrier.^[^ ^63^, ^64^ ^]^ However, the synthetic challenges and limited stability of RNA under prebiotic conditions, as well as the complexities of transitioning from an RNA‐only to an RNA–protein world in extant life, raise significant questions about its plausibility. Thus, it has recently been proposed that amyloids might have played a crucial role in prebiotic molecular evolution,^[^ ^65^, ^66^, ^67^, ^68^ ^]^ working alongside RNA in a mutually beneficial scenario.

**Figure 2:**
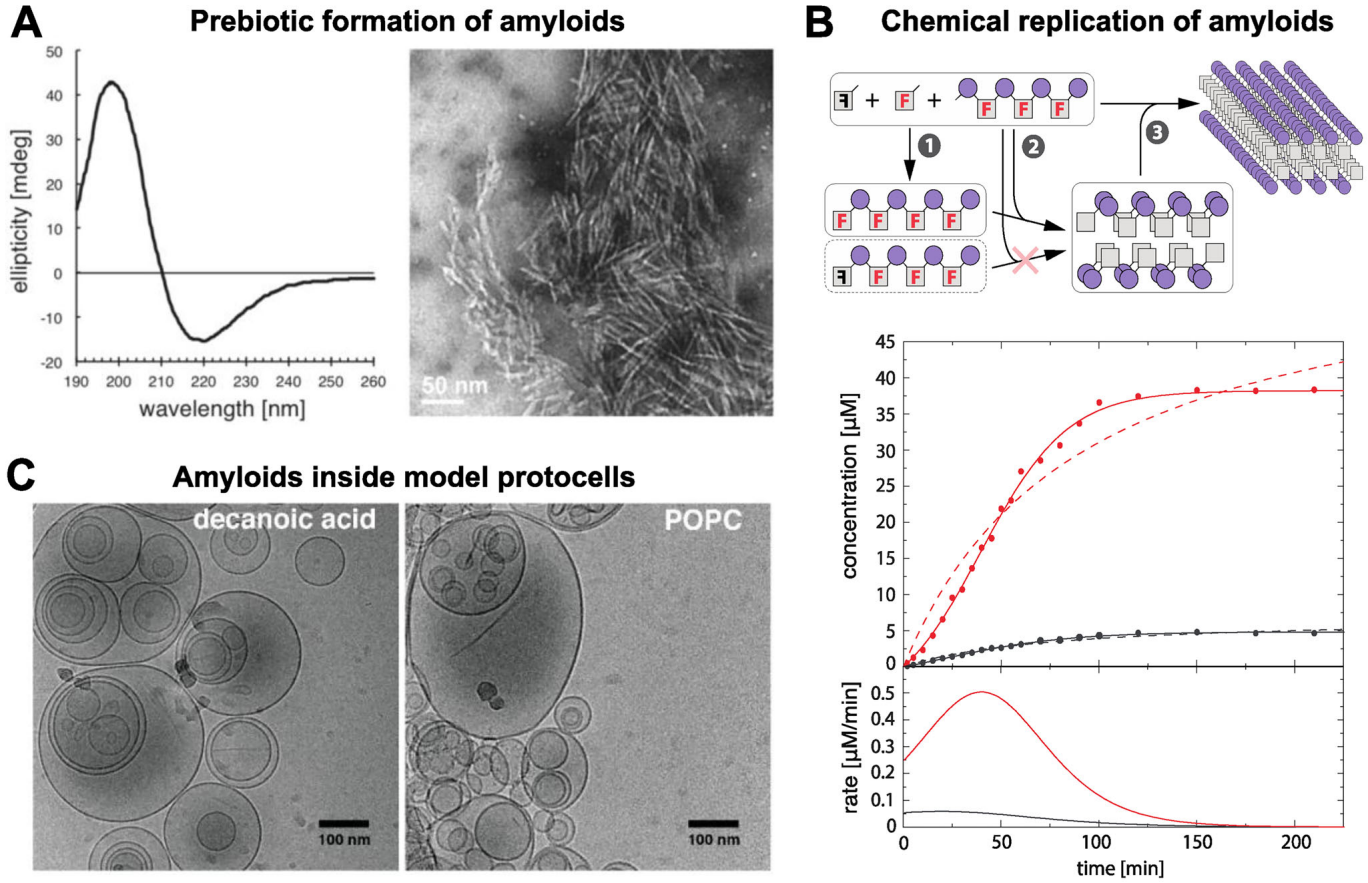
Peptide amyloids in the origin of life. A) CD spectroscopy of precipitates from a condensation reaction in water containing 8 × 10^−3^ m glycine, 8 × 10^−3^ m alanine, 3 × 10^−3^ m valine, and 1 × 10^−3^ m aspartate reveals characteristic β‐sheet‐structured aggregates. Negatively stained electron micrographs further confirm a fibril‐like morphology in the precipitates. Adapted with permission.^[^ ^70^ ^]^ Copyright 2016, John Wiley and Sons. B) A schematic illustrating the mechanism of amyloid‐templated autocatalytic condensation of activated amino acids. The black F and the mirror‐image red F denote the enantiomeric forms of the amino acid involved in the reaction. The plot below the scheme depicts the autocatalysis of the (FR)_4_ peptide amyloid in a reaction involving R(FR)_3_ and racemic phenylalanine (F:L‐phenylalanine and f: D‐phenylalanine). The time‐dependent product formation is shown for (FR)_4_ (red) and its diastereomer fR(FR)_3_ (gray), with solid and dashed lines representing fits to an autocatalytic and second‐order reaction mechanism, respectively. The lower plot displays the derivative of the autocatalytic fits from the upper plot. Adapted with permission.^[77]^ Copyright 2022, The Authors. C) Valine addition to V(DV)_4_ leads to amyloid formation within decanoic acid and POPC vescicles. Cryo‐electron micrographs show amyloid aggregates predominantly forming inside the vescicles, with only rare occurrences outside. Reproduced with permission.^[80]^ Copyright 2021, John Wiley and Sons.

Peptide amyloids, with their repetitive structure composed of intermolecular β‐strands, present an interesting candidate for the first replicative chemical entity with biological potential. Their minimal requirements concerning amino acid composition and peptide length, combined with unique properties such as exceptional stability and catalytic activity, make them highly relevant in this context. Under aqueous prebiotic conditions, the presence of carbonyl sulfide (COS) can activate amino acids, allowing them to condense into peptides.^[^ ^69^ ^]^ It has been demonstrated that amyloids can spontaneously form as precipitates through the COS‐mediated polymerization of prebiotic amino acids, such as valine, alanine, glycine, and aspartate (**Figure** **2**A).^[^ ^70^ ^]^ Another key feature of amyloids is their ability to serve as templates for replication. Amyloids can thus act as chemical templates in ligating shorter peptide fragments, including self‐correction facilitated by the cross‐β fold.^[^ ^71^, ^72^, ^73^ ^]^ In the prebiotic polymerization of amino acids, amyloid templating has been suggested as a mechanism for forming long isotactic peptides during the polymerization of racemic amino acids, as peptide isotacticity satisfies the stereochemical restraints of the β‐sheets.^[^ ^74^ ^]^ More recently, it was demonstrated that amyloids can promote template‐directed sequence‐selective, regioselective, and stereoselective sequential addition of amino acids to peptides, similar to the mechanisms of DNA/RNA replication/transcription.^[^ ^75^, ^76^ ^]^ Amyloids can also act as self‐replicators in an autocatalytic manner, enhancing the yield and stereoselectivity of their own formation (Figure 2B).^[^ ^77^ ^]^ Furthermore, there has been a growing body of reports on short peptides highly organized in an amyloid fold, which can resemble a protein‐like quaternary structure, enabling them to support various catalytic activities.^[^ ^66^, ^78^ ^]^

Due to their repetitive structures, both amyloids and other biologically relevant molecules, such as RNA/DNA, fatty acid bilayers and polysaccharides, can interact cooperatively, resulting in enhanced affinity through avidity. The cooperative interactions between amyloids and vesicle‐forming fatty acids can offer insights into forming early membranes.^[^ ^79^ ^]^ Furthermore, peptide amyloids have been shown to chemically replicate within membrane vesicles (Figure 2C).^[^ ^80^ ^]^ The aggregation of amyloidogenic peptides is also accelerated in the presence of polyphosphates^[^ ^81^ ^]^ and sugars like glycosaminoglycans or GAGs.^[^ ^82^ ^]^ Research on the cooperative assembly of amyloids and nucleic acids suggests a potential early connection between the peptide and RNA prebiotic worlds.^[^ ^83^, ^84^ ^]^ Very recently, the sequence‐selective interactions between amyloids and RNA triplets was demonstrated to address one of the most fundamental problems of biology: the origin and evolution of the primitive genetic code.^[^ ^18^ ^]^

The arguments discussed above strengthen the idea of the potential role of peptide amyloids in the origin of life and the hypothesis that the functional amyloid fold is the LUCA (Last Universal Common Ancestor) of protein folds.^[^ ^85^ ^]^ Indeed, the studies on the amylome indicate that most proteins have short segments that are amyloidogenic.^[^ ^86^, ^87^ ^]^ In addition, a statistical analysis of all known proteomes has shown that the amyloidogenic potential of peptide segments is higher than random expectation.^[^ ^88^ ^]^ The study demonstrated that amyloidogenic or aggregation‐prone peptide segments are overrepresented in extant proteomes compared to sequence‐randomized proteomes and that the archaeal proteomes contain more amyloidogenic sequence motifs than larger primate proteomes. This further suggests that as proteomes evolve to become larger and more complex, the relative abundance of amyloidogenic sequences decreases.^[^ ^89^, ^90^ ^]^ Thus, it is implied that the amyloid folds were more prevalent in evolutionary history than they are now and that they may have been involved in various biological processes, including translation, transcription or detoxification against metal oxides.^[^ ^91^ ^]^ Hence, functional amyloids are better understood as direct descendants of LUCA rather than as peculiar entities as they are often regarded. Also, the above‐hypothesized differentiation between functional and disease‐associated amyloids based on evolutionary optimization could be distilled to suggest that disease‐associated amyloid proteins may not have evolved sufficiently away from their ancestral amyloidogenic sequences, and thus their amyloid‐forming propensity could still be partly evolutionarily conserved, whereas functional amyloids are sequence‐optimized to assemble into fibrils in a regulated, physiologically beneficial manner.

Through this long evolutionary history, a wide array of organisms have maintained the use of functional amyloids. Many functional amyloids, as shown in **Table** **1**, are important to the organism's overall fitness.^[^ ^14^ ^]^ Bacterial functional amyloids, particularly in Enterobacteriaceae, are the most well‐studied. Studying these systems offers a unique perspective to learn how organisms have evolved to utilize functional amyloids through the development of tight regulation on protein aggregation.

**Table 1:** Representative functional amyloids across the domains of life and their respective functions.

Functional Amyloid	Organism	Function
Bacterial		
Curli (CsgA and CsgB)^[^ ^92^ ^]^	*Escherichia* and *Salmonella*	Biofilm matrix production
FapC^[^ ^93^ ^]^	*Pseudomonas*	Biofilm matrix production
TasA^[^ ^94^ ^]^	*Bacillus*	Biofilm matrix production
Phenol‐soluble modulins^[^ ^95^ ^]^	*Staphylococcus*	Biofilm matrix production
MTP^[^ ^96^ ^]^	*Mycobacterium*	Pilli formation
P1,^[^ ^97^ ^]^ WapA,^[^ ^98^ ^]^ SMU_63c^[^ ^98^ ^]^	*Streptococcus*	Biofilm matrix production
HpaG^[^ ^99^ ^]^	*Xanthomonus*	Pathogenicity factor
Microcin E492^[^ ^100^ ^]^	*Klebsiella*	Regulation of toxicity
Rodlins,^[^ ^101^ ^]^ Chaplins^[^ ^102^ ^]^	*Streptomycetes*	Reduce water surface tension
Archael		
MspA^[^ ^103^, ^104^ ^]^	Methanosaeta and Methanospirillum	Extracellular cell wall sheath formation
HVO_1403 (unconfirmed) ^[^ ^105^ ^]^	*Haloferax*	Biofilm matrix production
Eukaryotic		
REF^[^ ^106^ ^]^	*Hevea brasiliensis*	Natural rubber production
Chorion^[^ ^107^ ^]^	*Bombyx mori*	Silkmoth eggshell production
PMEL17^[^ ^24^ ^]^	*Homo sapiens*	Directs melanin polymerization
Spidroins^[^ ^108^ ^]^	*Araneae*	Spider silk formation

### 1.3 Curli Fibrils as a Model Functional Amyloid

**Figure 3:**
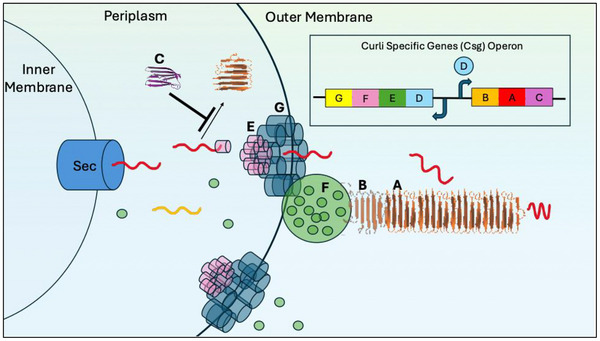
Curli fibril biogenesis. Curli fibril formation is controlled by seven different curli specific genes on two divergently transcribed operons. The curli fibril is made up of functional amyloid proteins CsgA^[^ ^122^ ^]^ and CsgB.^[^ ^123^ ^]^ CsgC functions as an amyloid chaperone, preventing the formation of periplasmic aggregates. CsgD is a transcriptional regulator of the CsgBAC operon. CsgE functions as an accessory chaperone aiding in efficient CsgA and CsgB secretion through the pore, CsgG. Finally, CsgF is hypothesized to function as an accessory protein that ensures the fibril is surface anchored. This regulation ensures that CsgA and CsgB are secreted in a soluble form to form the functional amyloid, curli, on the outer membrane.

The first described and most studied functional amyloid protein is CsgA from *E. coli*.^[^ ^92^ ^]^ CsgA is the major subunit of a cell‐surface attached filament called curli which are found on various Enterobacteriaceae.^[^ ^92^ ^]^ The minor subunit of the *E. coli* curli fibril is a nucleator protein called CsgB.^[^ ^109^ ^]^ Curli fibrils makeup the major proteinaceous component of the extracellular matrix that functions to protect bacterial biofilms from various stressors.^[^ ^110^, ^111^, ^112^ ^]^ As functional amyloid protein subunits, CsgA and CsgB production is heavily regulated during biofilm growth to prevent the formation of cytotoxic amyloid intermediates and fibrils.^[^ ^47^, ^109^, ^113^, ^114^, ^115^, ^116^, ^117^ ^]^ Bacteria can mitigate the toxicity associated with amyloid formation by secreting the curli subunits, CsgA and CsgB, as soluble protein monomers.^[^ ^109^, ^118^, ^119^ ^]^ The secretion and assembly of CsgA and CsgB into curli fibers is largely orchestrated by two divergently transcribed operons that encode 7 “Csg” proteins (**Figure** **3**). CsgA and CsgB are secreted out of the cell by the CsgG secretory pore.^[^ ^115^, ^117^, ^120^ ^]^ Once secreted, CsgB begins to fold into the canonical β‐sheet rich structure and creates a nucleus from which CsgA monomers add on.^[^ ^92^, ^109^, ^118^, ^119^, ^121^ ^]^ Along with an accessory protein, CsgF,^[^ ^116^ ^]^ this nucleation‐dependency regulates curli formation both spatially and temporally, ensuring that the fibril forms extracellularly and is anchored onto the outer cell membrane.

Two additional mechanisms aid in preventing periplasmic aggregation of CsgA and CsgB prior to their secretion. The first is chaperone‐based control.^[^ ^48^ ^]^ CsgC, a periplasmic chaperone, inhibits CsgA and CsgB amyloid formation at sub‐stoichiometric ratios by preventing the formation of an amyloid nucleus.^[^ ^47^, ^48^, ^124^, ^125^ ^]^ Interestingly, CsgC controls amyloid formation in the absence of ATP or any known hydrolysable energy source,^[^ ^48^, ^125^ ^]^ indicating that its amyloid control has uniquely evolved to function as a potent periplasmic amyloid inhibitor. The second mechanism is gatekeeper formation. Both CsgA and CsgB contain residues that electrostatically interact intramolecularly to slow amyloid formation, presumably also by blocking nucleus formation.^[^ ^126^, ^127^, ^128^, ^129^ ^]^

Humans possess a robust microbiota, consisting of archaea, fungi, bacteria, parasites, and viruses. We have co‐evolved alongside this microbiota, developing a delicate balance where most interactions between bacteria and their hosts are neutral or positively beneficial. Of course, some bacteria, including CsgA‐expressing *E. coli*, cause disease in humans and amyloids can be important bacterial virulence factors. Here, we discuss a variety of host‐microbial functional amyloids interactions and their consequences.

### 1.4 Functional Amyloids as a Tool for Host Cell Adhesion and Invasion

Prior to the discovery of curli subunits CsgA and CsgB as functional amyloids, the curli fibril was studied for its ability to adhere to surfaces. *E. coli* mutants that displayed increased adherent ability carried mutations leading to an increase in curli production that allowed for colonization of hydrophilic surfaces such as glass and hydrophobic surfaces such as polystyrene.^[^ ^130^ ^]^ A similar phenomenon was shown for the curli‐fibril produced by *S. enteriditis* and its ability to adhere to Teflon and stainless steel.^[^ ^131^ ^]^

In addition to abiotic surfaces, *E. coli* clinical isolates expressing curli also bind to fibronectin.^[^ ^109^, ^132^ ^]^ Fibronectin is found in human serum in its soluble form and in the extracellular matrix in an insoluble form.^[^ ^133^ ^]^ Curli binding to both the soluble or insoluble form of fibronectin may have consequences in the host‐pathogen interaction. Curliated *E. coli, S. enteriditis*, and *S. typhimurium* have higher adhesion and invasion into eukaryotic cells than do non‐curliated strains^[^ ^114^, ^134^, ^135^, ^136^ ^]^ and curli‐mediated invasion of HeLa cells is dependent on the presence of serum.^[^ ^137^ ^]^ Serum contains fibronectin in its soluble form^[^ ^133^, ^138^, ^139^ ^]^ suggesting that curli‐mediated host‐cell adhesion and invasion is linked to the ability of curli to bind soluble fibronectin.^[^ ^137^ ^]^ Curliated cells may also be able to bind insoluble fibronectin in the cellular matrix to enhance host cell adhesion.

Through an independent mechanism, curli expression can also lead to fibrinolysis, or the breakdown of fibrin. Curliated cells can bind plasminogen and tissue‐type plasminogen activator (t‐PA).^[^ ^140^ ^]^ Curli can then act as a scaffold, allowing t‐PA to interact with, and convert, plasminogen into plasmin.^[^ ^140^ ^]^ Plasmin is a fibrinolytic protein that degrades fibrin blot clots and breaks down soft tissue laminin, fibronectin, and collagen.^[^ ^141^ ^]^ Therefore, curliated cells may increase fibrinolysis and break down the extracellular matrix, allowing for increased bacterial dissemination and invasion of the host. While curli fibrils are inherently sticky, these mechanisms illustrate how curli fibrils have a direct and purpose‐driven role in both cellular adhesion and invasion by either 1) binding directly to fibronectin or 2) facilitating its breakdown through plasmin formation.

Increased bacterial internalization in a host cell can lead to the consequence of increased antigen presentation. When a bacterial pathogen is internalized, its cellular components can be degraded by the host cell and the resulting peptides will be presented by MHC‐I leading to T‐cell recognition.^[^ ^142^ ^]^ Interestingly, curli may be able to interrupt this process, aiding in the bacterium's success in host colonization. Some bacteria possess toxins deemed “superantigens” which they use to manipulate the host immune system.^[^ ^143^ ^]^ Previously, all superantigens were found to co‐opt MHC‐II to modulate the immune response. Curli, on the other hand, interacts specifically with MHC‐I, and not MHC‐II^[^ ^144^ ^]^ which leads to a variety of benefits for the bacterium. First, while MHC‐II is specifically found in designated antigen‐presenting cells, MHC‐I is found in all nucleated cells. The ability of curli or curliated cells to bind to MHC‐I may allow these bacterium to adhere and colonize this wide variety of MHC‐I expressing cells within the host.^[^ ^144^, ^145^ ^]^ Currently, there is not enough direct evidence to show that this increased adherence to MHC‐I expressing cells leads to increased bacterial internalization in vivo. However, binding of the curliated cell to MHC‐I may disrupt the canonical antigen‐MHC‐I presentation by blocking MHC‐I from binding to degraded components of internalized bacteria, hindering the recruitment of T‐cells during a bacterial infection. Therefore, this mechanism allows curli to act as a unique superantigen by manipulating the host immune system through the MHC‐I, potentially leading to increased bacterial internalization through an increase in bacterial‐host cell adhesion. The natural consequence of increased bacterial internalization may also be avoided by blocking MHC‐I antigen‐presentation, however, more research is needed to confirm if this mechanism is utilized in vivo.

### 1.5 Functional Amyloids Are Pro‐inflammatory in the Host Leading to Increased Bacterial Dissemination

Curli fibril's ability to break down fibrin may also aid in cellular invasion by increasing host inflammation. Curli fibrils from *E. coli* and *S. pyogenes* have been found to bind to factor XI, factor XII, and pre‐kallikrein.^[^ ^146^, ^147^ ^]^ Previously, we described how curli is able to form plasmin by acting as a scaffold bringing together plasminogen and t‐PA, ultimately leading to fibrinolysis.^[^ ^140^ ^]^ In a similar fashion, the curli fibril brings together factor XII and pre‐kallikrein enabling the activation and formation of kallikrein,^[^ ^146^, ^147^ ^]^ a serine‐protease heavily involved in both clotting and fibrinolysis. This mechanism modulates host blood pressure and inflammation leading to increased tissue permeability and bacterial invasion.^[^ ^148^ ^]^ Following the pattern described so far, *E. coli* and *S. pyogenes* curli fibrils are also able to bind to H‐kininogen.^[^ ^146^, ^147^ ^]^ Kallikrein plays a crucial role in releasing bradykinin from H‐kininogen.^[^ ^148^ ^]^ Bradykinin is pro‐inflammatory and triggers the release of nitric oxide. This overproduction of nitric oxide increases in a time‐dependent manner when a host is infected with curli‐expressing bacteria leading to hypotension, vasodilation, and increased tissue permeability.^[^ ^149^ ^]^ Finally, curliated cells are also detected by TLR‐2 due to CsgA/CsgB acting as a pathogen‐associated molecular pattern.^[^ ^150^ ^]^ This recognition leads to the expression of pro‐inflammatory cytokines, TNF‐α, IL‐6, and IL‐8 which leads to further exacerbation of host vasodilation and fluid accumulation.^[^ ^151^ ^]^ This systemic exacerbation of the host inflammatory system likely aids in widespread bacterial dissemination throughout the host and may explain why curli‐overproducing cells lead to faster mortality in mouse models in a dose‐dependent manner.^[^ ^152^ ^]^

**Figure 4:**
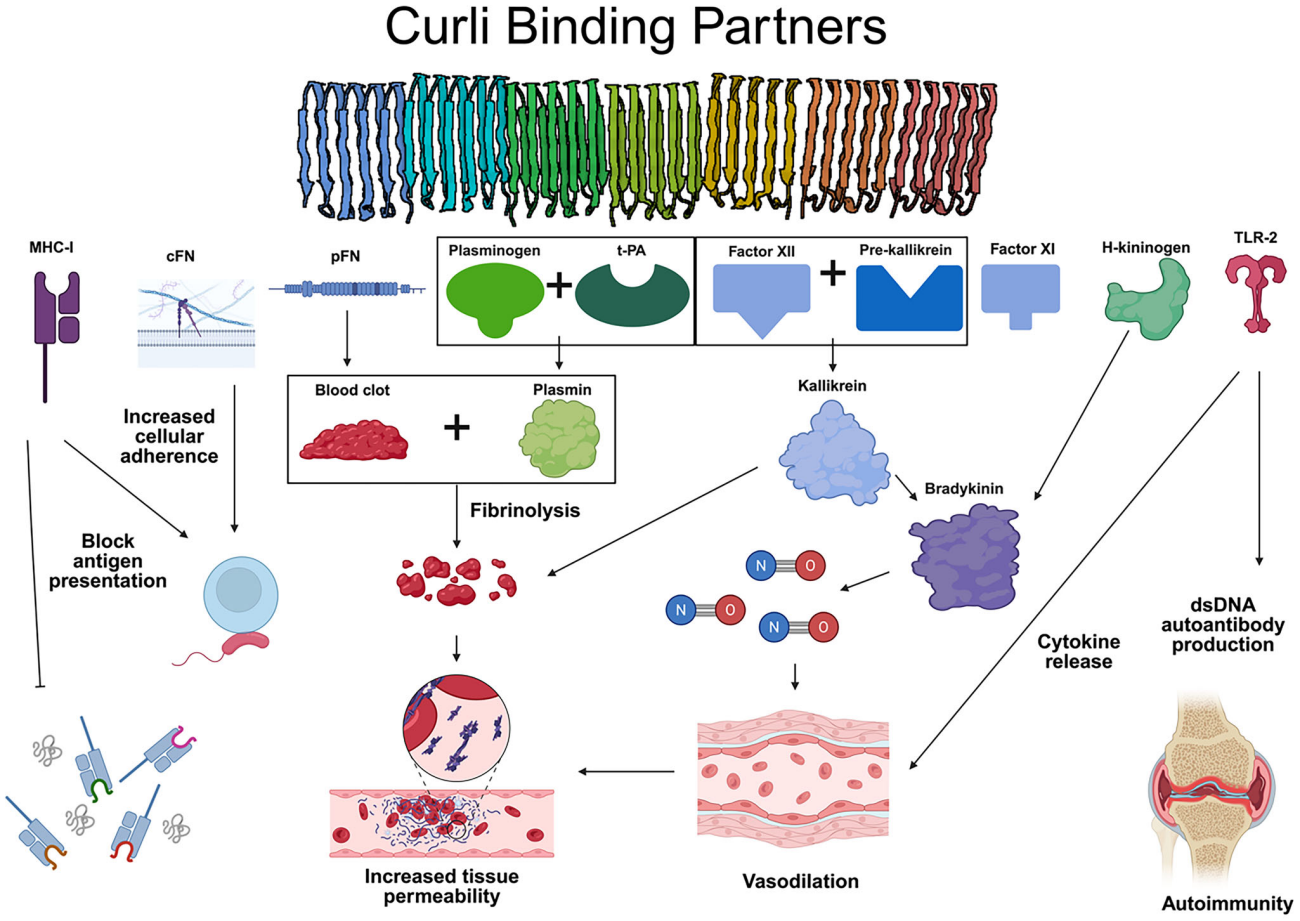
Curli binding partners and their role in host pathogenesis. Curli fibrils have multiple host protein binding partners that may play a role in exacerbation of the infection. These mechanisms include 1) bacterial adhesion to host cells in the case of MHC‐1 and cFN 2) block or exacerbate proper immune function in the case of MHC‐1 and TLR‐2, and 3) increase tissue permeability for bacterial dissemination in the case of pFN, plasminogen, t‐PA, factor XII, pre‐kallikrein, H‐kininogen, and TLR‐2. All three of these mechanisms require the presence of amyloid comprised of either CsgA and/or CsgB to act as either a scaffold or to enhance binding avidity. CsgA PDB ID: 8ENQ.^[^ ^157^ ^]^

These curli‐mediated effects not only lead to systemic inflammation and increased bacterial dissemination^[^ ^153^ ^]^ but can also lead to localized auto‐immunity. Gastrointestinal infections with *S. typhimurium* or *E. coli* producing curli fibrils lead to the development of autoimmunity following the formation of dsDNA autoantibodies, joint inflammation, and potentially triggering anti‐amyloid antibody formation.^[^ ^154^, ^155^, ^156^ ^]^ This autoantibody generation is catalyzed by CsgA/CsgB detection by TLR2 and potentially TLR9.^[^ ^156^ ^]^ This autoinflammatory reaction leads to bone reabsorption and reactive arthritis, and has been shown to contribute to systemic lupus erythematosus (SLE).^[^ ^155^, ^156^ ^]^ Taken together, bacterial functional amyloids not only manipulate the host immune and clotting system to enhance bacterial adhesion, invasion, and dissemination, but can also lead to systemic and long‐term health effects as summarized in **Figure** **4**.

### 1.6 Bacterial Functional Amyloids May Play a Role in the Development of Neurodegenerative Diseases

Perhaps the most interesting consequence of uncontrolled bacterial dissemination, as it pertains to functional amyloids, is the development of neurodegeneration in the host. This budding field was recently coined “mapranosis,” or the study of microbiota‐associated proteopathy and inflammation, in 2017 by Friedland and Chapman.^[^ ^158^ ^]^ The term mapranosis, while not commonly used in literature, is one facet of the long studied field of host–microbe interactions. Mapronosis currently focuses on the link between the microbiota and the development of neurodegenerative diseases such as Alzheimer's disease and Parkinson's disease. We initially discussed the structure and evolution of amyloids which is both unique and conserved. While functional and pathogenic amyloids have some distinct differences, this structural conservation between functional and disease associated amyloids is the first big hint as to how bacterial amyloids may cause neurodegeneration: molecular mimicry.^[^ ^159^ ^]^

Many bacteria present in the human microbiome utilize functional amyloids, including *Streptococcus*,^[^ ^160^ ^]^ *Staphylococcus*,^[^ ^161^ ^]^ *Klebsiella*,^[^ ^162^ ^]^ *Citrobacter*,^[^ ^163^ ^]^ *Escherichia*,^[^ ^92^ ^]^ *Bacillus*,^[^ ^164^ ^]^ *Mycobacteria*,^[^ ^165^ ^]^ and *Salmonella* ^[^ ^131^ ^]^ among others. A plethora of evidence has shown that patients with cognitive impairment display dysbiosis, along with an increase in these taxa, and display increased pro‐inflammatory markers.^[^ ^166^, ^167^, ^168^, ^169^, ^170^, ^171^ ^]^ We have previously described how the presence of amyloid‐producing bacteria within the host leads to an increased immune response. Interestingly, the immune response mounted against the bacterial functional amyloid has been linked to autoimmunity to host amyloidogenic proteins, which may exacerbate the symptoms of neurodegeneration.^[^ ^159^, ^166^, ^172^, ^173^ ^]^ However, this does not explain how bacterial functional amyloids may be involved in the initial development of amyloid‐related neurodegeneration.

Multiple studies have shown that the presence of curli‐producing bacteria within the gut^[^ ^174^ ^]^ increases motor defects in organisms that did not initially display amyloid aggregates in the brain.^[^ ^169^, ^172^, ^175^, ^176^ ^]^ One property of disease‐associated amyloids is the ability to self‐propagate in a prion‐like fashion which leads to increased amyloid formation and transmission.^[^ ^15^, ^17^, ^177^ ^]^ Bacterial amyloids that display molecular mimicry to pathogenic amyloids may jump‐start this process of self‐propagation through a process called “seeding.” Seeding has been best shown in the interaction between *P. aeruginosa* FapC^[^ ^178^ ^]^ and human amyloid‐β protein (associated with Alzheimer's disease) and the interaction between multiple homologs of CsgA^[^ ^179^ ^]^ and human alpha‐synuclein (associated with Parkinson's disease) and human Amyloid A amyloidosis. Both FapC^[^ ^178^ ^]^ and CsgA^[^ ^175^, ^180^ ^]^ have been found to accelerate their respective human amyloid protein in vitro in a similar fashion. In both cases, reducing the amyloidogenicity of FapC and CsgA by slowing their intrinsic ability to form amyloid increases their ability to seed and accelerate human amyloid in vitro.^[^ ^179^, ^181^ ^]^ Thus far, there has been no direct observation of the initial physical interaction and acceleration of human amyloid by functional amyloid leading to the development of amyloid deposition in vivo. While it is unknown if this mechanism occurs in vivo, alpha‐synuclein aggregates have been found in the gut in patients in the early stages of Parkinson's disease^[^ ^182^, ^183^ ^]^ and injection of alpha‐synuclein seeds into the gut is capable of promoting brain pathology associated with Parkinson's disease.^[^ ^184^ ^]^ In addition, oligomers and seeds of FapC, alpha‐synuclein, and amyloid β have been shown to induce endothelial leakiness.^[^ ^185^ ^]^ This leakiness is due to binding of the anionic amyloid seed or oligomer to VE‐cadherin, leading to actin remodeling and downregulation of tight‐junction proteins.^[^ ^185^ ^]^ Altogether, anionic amyloid oligomer and seeds led to an increase in gap formation and vascular leakiness in human microvascular endothelial cells, human brain endothelial cells, and in mice, indicating a potential route for functional and disease associated amyloid intermediates to bypass the gut and subsequently the blood brain barrier.^[^ ^185^ ^]^ Altogether, this research supports the hypothesis that human amyloids within the gut may be seeded by gut bacterial amyloids,^[^ ^186^ ^]^ potentially leading to deposits of aggregates within the brain through transneuronal propagation^[^ ^187^ ^]^ or by dissemination of amyloid intermediates caused by inflammation and a disrupted gut epithelial layer.^[^ ^135^, ^136^, ^153^, ^184^, ^185^ ^]^

## 2 Conclusion

In this review, we covered the structural characteristics of amyloid proteins and the distinctions between functional and disease associated amyloids. We connect the self‐replicative nature of amyloids to a potential role in the origin of life and propose that the amyloid fold may be the LUCA of protein folds. Finally, we discuss the evolutionary conservation of the amyloid fold throughout the domains of life and highlight how functional amyloids are utilized by bacteria to colonize a host. Ultimately, we highlight how the structural characteristics of amyloids unveils their importance in both functional and disease‐associated biology. Characterization of new bacterial functional amyloids will greatly enhance our understanding of amyloid structure and control, amyloid evolution, and amyloid utilization which can have profound impacts in fields studying autoimmunity, neurodegeneration, and bacterial‐host interactions. In particular, the connection between functional amyloids and neurodegeneration has only just begun to be studied. Future work must include visualizing cross‐seeding and amyloid intermediate transport in vivo to better understand where and how functional amyloids may jumpstart amyloid formation within the host. In addition, more work must be done to characterize the morphology of disease‐associated amyloids cross‐seeded by functional amyloids to determine if these fibrils are heterologous to previously studied polymorphs. This work could inform how cross‐seeding and molecular mimicry can affect the generation and toxicity of the disease‐associated amyloid and may open new avenues to understanding the causative agents of amyloid‐based diseases.
